# WRF-Chem modeling of particulate matter in the Yangtze River Delta region: Source apportionment and its sensitivity to emission changes

**DOI:** 10.1371/journal.pone.0208944

**Published:** 2018-12-07

**Authors:** Nan Li, Yilei Lu, Hong Liao, Qingyang He, Jingyi Li, Xin Long

**Affiliations:** 1 Jiangsu Key Laboratory of Atmospheric Environment Monitoring and Pollution Control, Jiangsu Collaborative Innovation Center of Atmospheric Environment and Equipment Technology, School of Environmental Science and Engineering, Nanjing University of Information Science & Technology, Nanjing, China; 2 Nanjing Gaochun district Meteorological Bureau, Nanjing, China; 3 Nanjing Star-jelly Environmental Consultants Co., Ltd, Nanjing, China; 4 Key Lab of Aerosol Chemistry & Physics, Institute of Earth Environment, Chinese Academy of Sciences, Xi’an, China; National Dong Hwa University, TAIWAN

## Abstract

China has been troubled by high concentrations of fine particulate matter (PM_2.5_) for many years. Up to now, the pollutant sources are not yet fully understood and the control approach still remains highly uncertain. In this study, four month-long (January, April, July and October in 2015) WRF-Chem simulations with different sensitivity experiments were conducted in the Yangtze River Delta (YRD) region of eastern China. The simulated results were compared with abundant meteorological and air quality observations at 138 stations in 26 YRD cities. Our model well captured magnitudes and variations of the observed PM_2.5_, with the normal mean biases (NMB) less than ±20% for 19 out of the 26 YRD cities. A series of sensitivity simulations were conducted to quantify the contributions from individual source sectors and from different regions to the PM_2.5_ in the YRD region. The calculated results show that YRD local source contributed 64% of the regional PM_2.5_ concentration, while outside transport contributed the rest 36%. Among the local sources, industry activity was the most significant sector in spring (25%), summer (36%) and fall (33%), while residential source was more important in winter (38%). We further conducted scenario simulations to explore the potential impacts of varying degrees of emission controls on PM_2.5_ reduction. The result demonstrated that regional cooperative control could effectively reduce the PM_2.5_ level. The proportionate emission controls of 10%, 20%, 30%, 40% and 50% could reduce the regional mean PM_2.5_ concentrations by 10%, 19%, 28%, 37% and 46%, respectively, and for places with higher ambient concentrations, the mitigation efficiency was more significant. Our study on source apportionment and emission controls can provide useful information on further mitigation actions.

## Introduction

Fine particulate matter (PM_2.5_) pollution in China has drawn sustained attention in recent years [1–6]. Human exposure to high PM_2.5_ can lead to adverse health impacts on cardiovascular and respiratory system [7–9]. Light extinction components in PM_2.5_ can seriously impair visibility, regarded as the dominant contributors to regional haze [6, 10–13]. In addition, PM_2.5_ can influence climate, via the direct effect of scattering/absorbing radiation and the indirect effect of changing cloud properties [14–16]. The Yangtze River Delta (YRD) region is one of the most PM_2.5_ polluted regions in China [17–20]. The annual mean PM_2.5_ concentration averaged for the YRD region is 70 μg m^-3^ in 2013, 100% higher than the national grade II standard at 35 μg m^-3^. Due to the continuous improvement efforts of local environmental authorities in recent years, the anthropogenic PM_2.5_ emissions has been reduced by ~30% [21], which significantly result in the PM_2.5_ concentration decreasing (~30%) during 2013 to 2017. Even so, more than 90% of the YRD cities still failed to meet the guideline.

PM_2.5_ pollution is jointly affected by meteorology and pollutant emissions [16,22]. Some typical synoptic situations improve the air quality, such as high mixing layer, strong wind and rich precipitation, in favor of diffusing or depositing the PM_2.5_ [23], but this is uncontrollable. We would pay more attention to source apportionment and emission control for the effective atmospheric environmental governance. Chemistry model is a useful tool to bridge the pollutant emissions with concentrations [24–30]. For instance, an updated CMAQ model was employed to simulate the PM_2.5_ in China for the whole year of 2013, using a newly developed inventory of anthropogenic emissions (MEIC) [31]. They proved the ability of the CMAQ model to reproduce the severe air pollution in China using the MEIC inventory, and emphasized the risk of human exposure to high level PM_2.5_. Also using CMAQ, the server haze pollutions occurred in eastern and central China in January 2013 were successfully reproduced in consideration of heterogeneous chemistry in secondary PM_2.5_ formation [32]. Focused on the sources of PM_2.5_, many model studies e.g. [31,33,34] agreed that local industrial and residential emissions as well as external transport considerably contributed to regional PM_2.5_ pollution episodes. In addition to the general sources, some studies e.g. [35] pointed out that ship emissions could contribute up to 11% to the PM_2.5_ concentrations along the shoreline of Bohai Rim region of China. The chemistry model was also applied to explore the potential impacts of emission control measures on PM_2.5_ reduction. For example, in the Guanzhong basin, estimated by WRF-Chem model, a 50% reduction of residential emissions could reduce the winter surface black carbon particles by up to 25% [25]. During the Second World Internet Conference in Hangzhou, the implemented emission control measures could effectively reduce the PM_2.5_ level by 7–25%, based on simulating results [36].

In this study, we employed a regional chemical model WRF-Chem to simulate PM_2.5_ concentrations in the YRD region for the year of 2015. We drove the simulation using the best currently available emission inventory of anthropogenic emissions. We evaluated the model performance by comparing simulated results with meteorological and air quality observations from 138 stations in 26 YRD cities. The aim of our study is to identify the source contributions from individual sectors and from different regions to PM_2.5_ concentration in the YRD region, and to explore the potential impacts of varying degrees of emission controls on PM_2.5_ reduction.

## Method and data

### Model

A specific version of the WRF-Chem model (Weather Research and Forecast model coupled with chemistry module, [37,38]) was applied to simulate PM_2.5_ pollution in the YRD region. This version of the model was developed by Li et al., [39–42], including updated gas-phase chemical [39,41], photochemistry [40], and aerosol modules [42,43]. This model has been applied to simulate PM_2.5_ and O_3_ pollutions in China multiple times and its performance was proved to be satisfactory e.g. [44,45].

**Fig 1:**
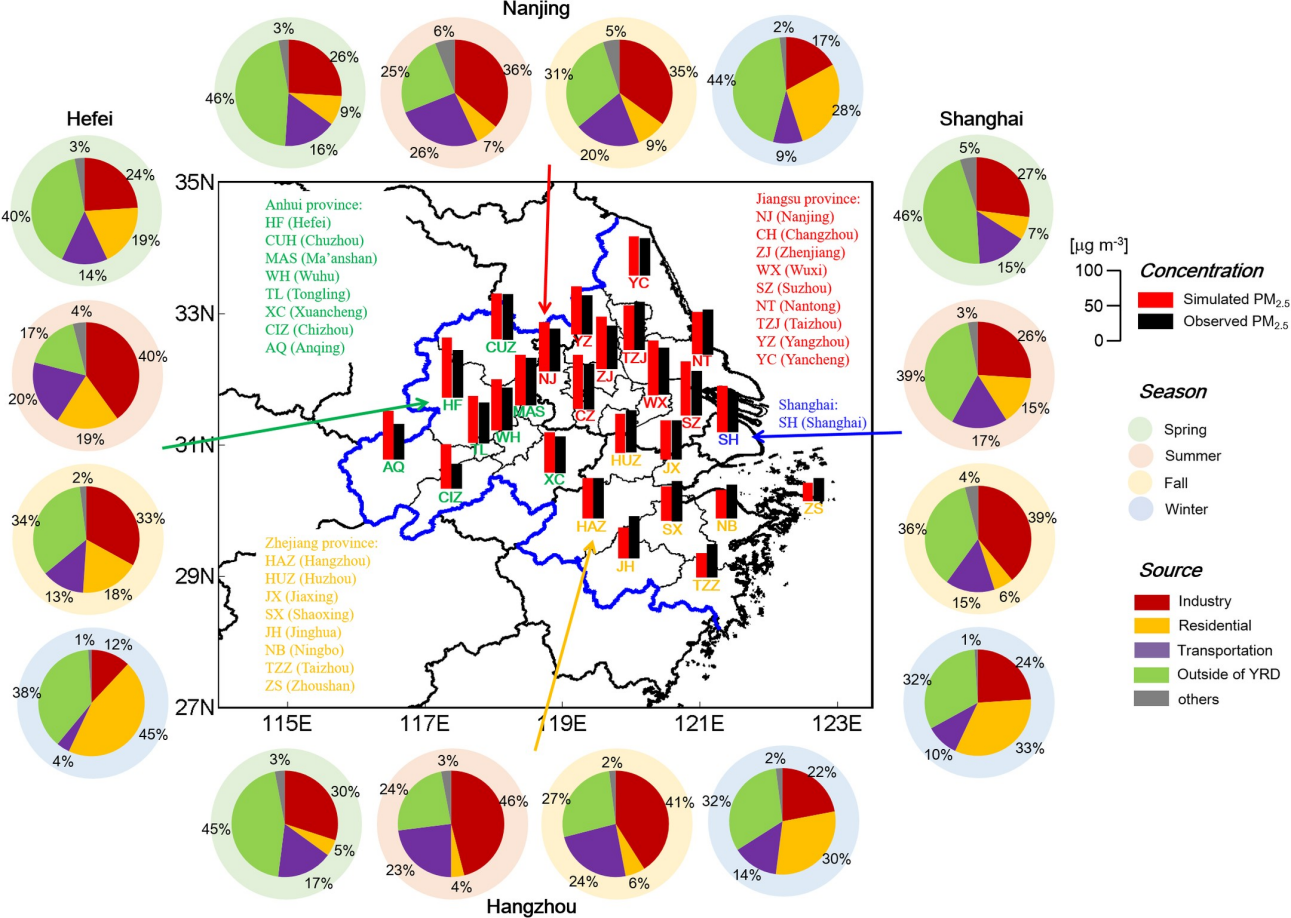
The simulation domain and the locations of the 26 cities in the YRD region. The area of bold blue line indicates the YRD region. The red and black bars are the concentrations of simulated and observed PM_2.5_ in each YRD city. The pie charts show sources of the simulated seasonal PM_2.5_ in four typical cities (Shanghai, Nanjing, Hangzhou and Hefei), including the relative contributions of local industry, transportation and residential sources, transport from outside of the YRD region, and other sources (power plants and open biomass burning).

We set two nested domains with horizontal resolutions of 36 km and 12 km, respectively, and the inner domain covered the YRD region (see Fig 1). Vertical layers extended from the surface to 50 hPa (28 layers), with 7 layers in the bottom one km to emphasize boundary layer processes. The meteorological initial and boundary conditions were derived by the National Centers for Environmental Prediction (NCEP) FNL Operational Global Analysis data (https://rda.ucar.edu/datasets/ds083.2/, last access: 18 November, 2018). The chemical initial and boundary fields were provided by a global chemical model (MOZART, Model for Ozone and Related chemical Tracers) [46]. We conducted four month-long simulations (January, April, July and October) to represent the typical meteorological and air conditions for each of the four seasons in 2015. Each month-long simulation was initialized for the last five days of the previous month.

### Emissions

Pollutant emissions, including anthropogenic, biogenic and open biomass burning emissions, are important inputs for chemistry simulation. We obtained anthropogenic emissions from the Multi-resolution Emission Inventory for China (MEIC) developed by Tsinghua University (http://www.meicmodel.org, [21]). MEIC is the most updated available emission inventory for China, which included monthly emissions from industry, power generation, transportation, residential and agricultural activities, and had a native resolution of 0.25°. We averaged the MEIC emission estimates for the years of 2014 and 2016 to represent that for 2015, because the data for 2015 is not available at present. S1 Fig shows the spatial distributions of anthropogenic SO_2_, NO_x_, VOCs and PM_2.5_ emissions in the YRD region. Anthropogenic actives strongly contributed to emissions in urban areas of central YRD, such as Zhenjiang, Changzhou, Wuxi, Suzhou and Shanghai.

Biogenic emissions were calculated by the Model of Emissions of Gas and Aerosols from Nature (MEGAN, [47]). The MEGAN is coupled into the WRF-Chem model, and provides on-line calculation of biogenic emissions. To drive MEGAN, the leaf area index (LAI), the plant function types (PFTs) and emission factors (EFs) are needed. The LAI and PFTs are based on Moderate Resolution Imaging Spectroradiometer (MODIS) products [48], and the EFs are taken from dominant species from Guenther et al. [47]. Open biomass burning emissions were quantified by the Fire INventory from NCAR (FINN, https://www2.acom.ucar.edu/modeling/finn-fire-inventory-ncar, last access: 18 November, 2018), which is based on satellite observations of active fires and land cover [49,50].

### Surface PM_2.5_ and meteorological observations

To a thorough evaluation of model performance, the air quality and meteorological observations with sufficient spatiotemporal coverage and resolution are need. We obtained surface hourly PM_2.5_ observations from 134 stations in 26 cities over the YRD region in January, April, July and October 2015 from the publishing website of China National Environmental Monitoring Center (http://datacenter.mep.gov.cn, last access: 18 November, 2018). Locations of all these cities can be found in Fig 1. The bars corresponding to each city in Fig 1 indicate the observed annual mean PM_2.5_ concentration, which was 57.6 μg m^−3^ averaged for the YRD region and exhibited remarkable variations in sub-regions. The Jiangsu province was the most polluted area, where the averaged PM_2.5_ concentration reached 62.4 μg m^−3^. The Zhejiang province was relatively less polluted with the averaged PM_2.5_ concentration of 52.6 μg m^−3^. Even more noteworthy is that all the cities exceeded the national grade II standard of PM_2.5_ at 35 μg m^-3^, except the island city Zhoushan in Zhejiang province.

We collected meteorological data with temperature, relative humidity and wind involved at 4 surface stations in Shanghai, Nanjing, Hangzhou and Hefei (see Table 1) (http://www.meteomanz.com, last access: 18 November, 2018). Meteorological conditions were generally mild and humid over the YRD region, with the annual mean temperature of 16.8–18.2°C and the relative humidity of 63–78%.

**Table 1:** The simulated and observed meteorological parameters in the YRD region.

	Mean		r	NMB	RMSE
	Simulated	Observed			
**Shanghai** (31.42°N, 121.45°E)					
Temperature (°C)	17.5	17.1	0.95	2%	2.7
Relative humidity (%)	78	70	0.71	11%	18
Wind speed (m s^-1^)	3.7	2.8	0.46	33%	1.8
**Nanjing** (31.93°N, 118.90°E)					
Temperature (°C)	16.8	16.5	0.96	6%	2.6
Relative humidity (%)	70	71	0.67	-1%	19
Wind speed (m s^-1^)	3.9	2.7	0.39	48%	2.2
**Hefei** (31.78°N, 117.30°E)					
Temperature (°C)	18.2	16.9	0.96	8%	2.9
Relative humidity (%)	63	72	0.65	-13%	21
Wind speed (m s^-1^)	3.2	2.1	0.50	52%	1.8
**Hangzhou** (30.23°N, 120.17°E)					
Temperature (°C)	18.2	17.6	0.94	3%	2.9
Relative humidity (%)	73	71	0.71	3%	18
Wind speed (m s^-1^)	2.8	2.3	0.35	25%	1.6

### Mitigation efficiency

In this study, we would explore the potential impacts of emission controls on PM_2.5_ reduction later. To better quantify the effects of different emission reduction schemes, we defined mitigation efficiency (ME) as the PM_2.5_ reduction ratio divided by the emission reduction ratio, as shown in Eq (1).

$$ME=\frac{(C_{0}-C_{1})/C_{0}}{(E_{0}-E_{1})/E_{0}}$$ (1)

where, ME is the mitigation efficiency, E_0_ is the initial anthropogenic emission, E_1_ is the anthropogenic emission in certain emission control scenario, C_0_ and C_1_ are the simulated PM_2.5_ concentrations using the emission E_0_ and E_1_, respectively.

## Results

### Model evaluation

Hourly observations of meteorology at 4 sites (Table 1) and PM_2.5_ at 134 sites (Table 2) were compared with the simulated results for validation.

**Table 2:** The simulated and observed PM_2.5_ concentrations in the YRD region.

City	Mean (μg m^-3^)		r	NMB	RMSE
	Simulated	Observed			
**Shanghai**	**65.4**	**57.7**	**0.51**	**13%**	**43.0**
Nanjing	70.7	61.2	0.50	16%	43.2
Changzhou	76.4	65.0	0.51	18%	46.7
Zhenjiang	74.5	62.0	0.50	20%	42.7
Wuxi	77.3	66.9	0.49	16%	44.2
Suzhou	77.5	64.1	0.45	21%	45.8
Nantong	60.7	63.8	0.51	-5%	43.4
Taizhou	63.7	69.2	0.56	-8%	42.3
Yangzhou	69.1	55.9	0.51	24%	41.4
Yancheng	55.8	53.4	0.56	4%	39.5
**Jiangsu average**	**69.5**	**62.4**	**0.51**	**11%**	**43.3**
Hefei	85.3	68.2	0.49	25%	48.6
Chuzhou	66.4	65.2	0.41	2%	43.3
Ma'anshan	70.8	68.0	0.48	4%	42.2
Wuhu	72.7	60.6	0.46	20%	42.4
Tongling	66.9	58.1	0.44	15%	39.9
Xuancheng	57.6	52.4	0.41	10%	41.2
Chizhou	64.0	35.4	0.42	81%	44.3
Anqing	67.5	50.6	0.40	33%	38.4
**Anhui average**	**68.9**	**57.3**	**0.45**	**20%**	**42.6**
Hangzhou	57.6	57.8	0.46	0%	36.7
Huzhou	55.4	60.7	0.42	-9%	41.9
Jiaxing	56.2	56.1	0.50	0%	36.1
Shaoxing	49.4	57.4	0.47	-14%	37.8
Jinhua	43.7	59.4	0.36	-27%	43.4
Ningbo	40.8	48.5	0.50	-16%	36.8
Taizhou	35.1	47.5	0.55	-26%	32.8
Zhoushan	26.3	33.0	0.57	-20%	26.5
**Zhejiang average**	**45.6**	**52.6**	**0.50**	**-13%**	**36.8**
**YRD average**	**61.8**	**57.6**	**0.49**	**7%**	**41.2**

**Fig 2:**
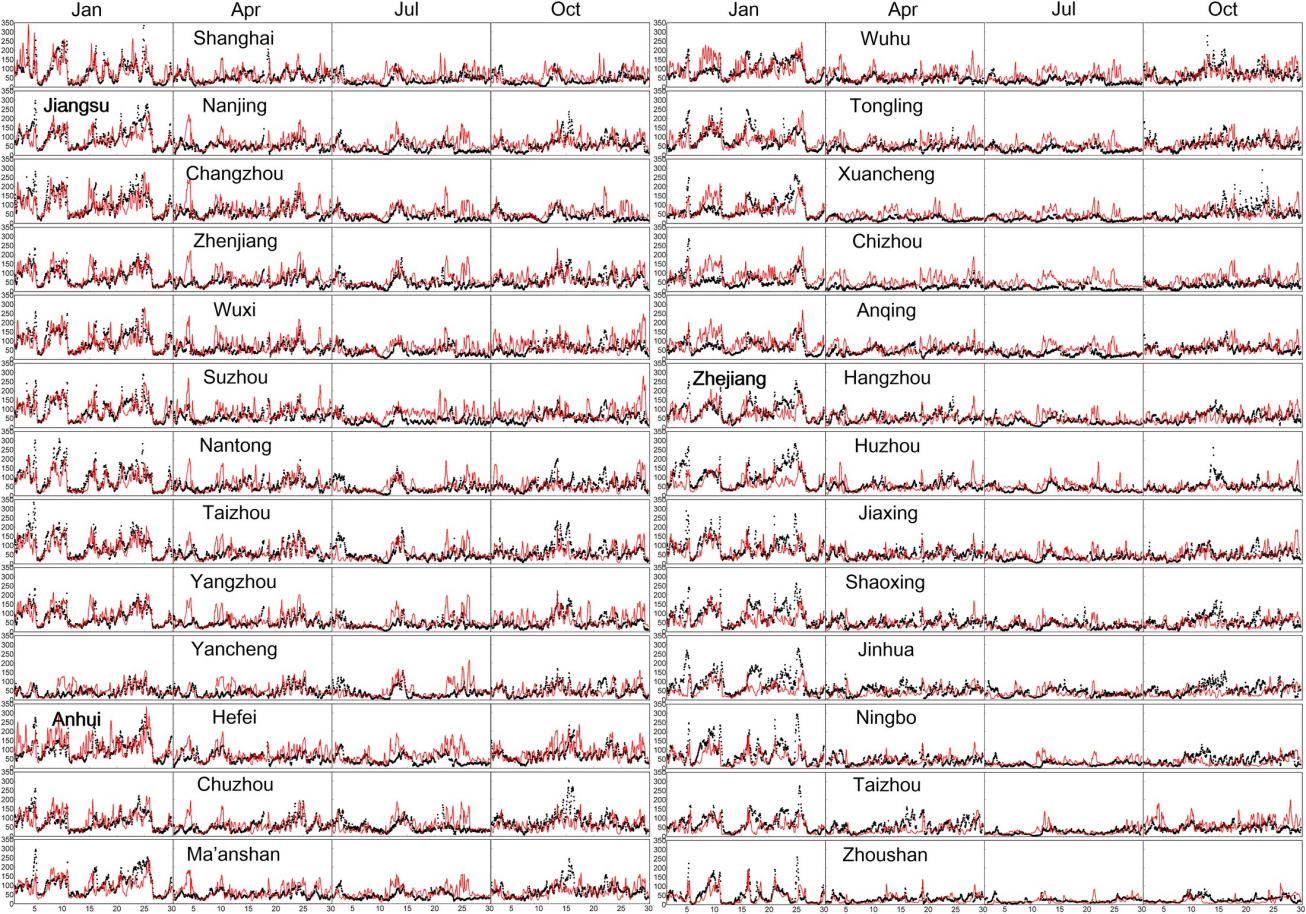
The simulated (red) and observed (black) PM_2.5_ concentrations (μg m^-3^) in 26 cities over the YRD region.

Fig 1 compares the simulated and observed annual mean PM_2.5_ concentrations at each of the 26 YRD cities. We averaged the grid concentrations matching at individual observation station level in each city to represent the city’s value. The simulated annual mean PM_2.5_ concentration was 61.8 μg m^−3^ averaged for the YRD region, 7% higher than the observed 57.6 μg m^−3^ (see Fig 1 and Table 2). Fig 2 shows the simulated and observed hourly PM_2.5_ concentrations in each of the 26 YRD cities. The seasonal mean simulated PM_2.5_ was the highest in winter and the lowest in summer, consistent with the observations. On the scale of individual cities, our model well reproduced the observed PM_2.5_ concentrations in most cities, with the normal mean biases (NMB) less than ±20% for 19 out of the 26 cities (Table 2). However, the model systematically overestimated the observed PM_2.5_ in Shanghai (NMB = 13%), Jiangsu (NMB = 11%) and Anhui (NMB = 20%). The overestimation has also been found in other simulation studies in China, but our difference was relatively smaller compared with the previous results [51], which is partly because our study used the latest emission inventory. In addition, our model incidentally overestimated the summertime PM_2.5_ in Shanghai, Jiangsu and Zhejiang (see Fig 2), mainly because of the omission of precipitation prediction.

**Fig 3:**
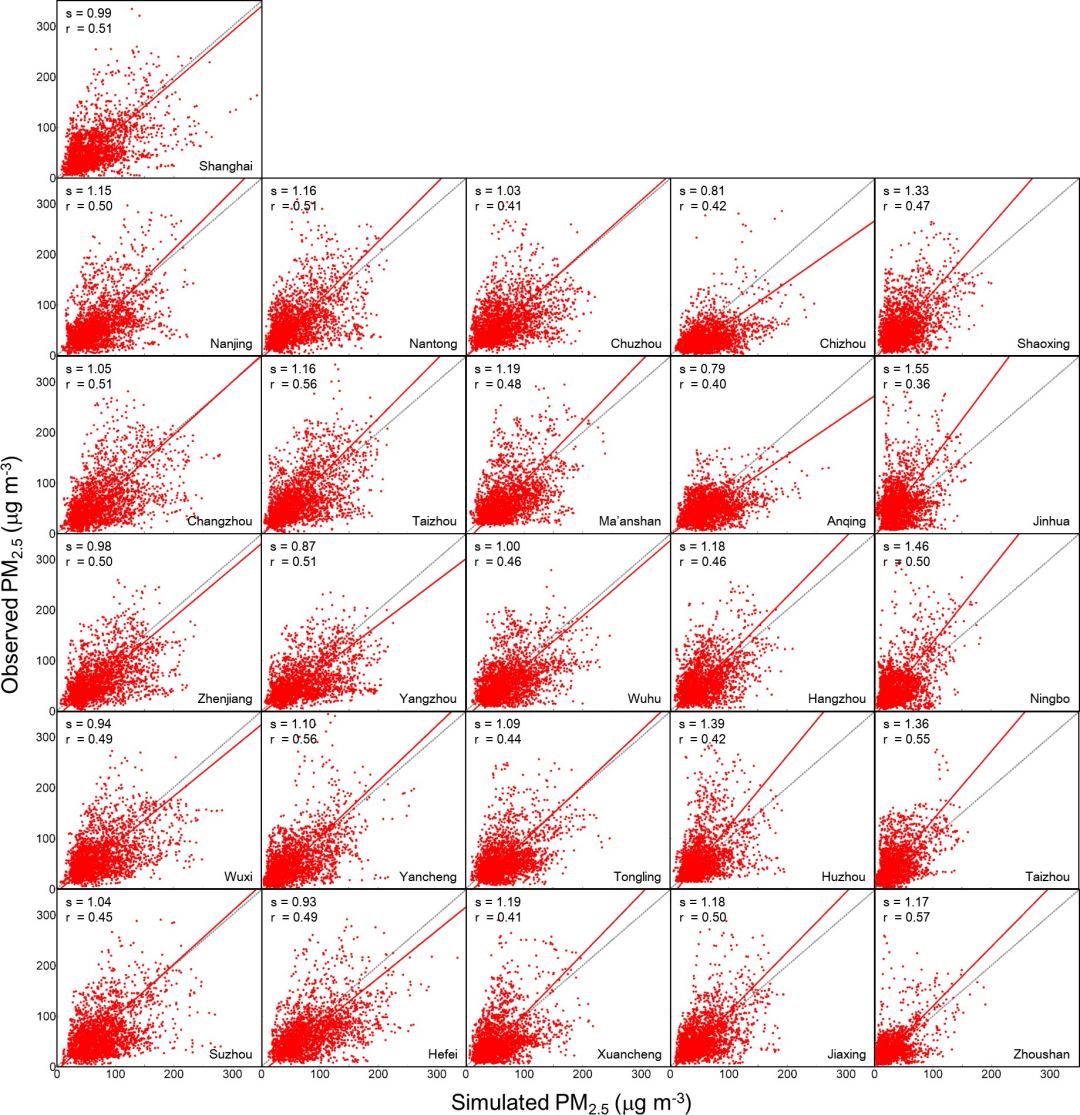
Scatter plots of simulated versus observed hourly PM_2.5_ concentrations at the 26 cities in the YRD region. Also shown are the reduced-major axis regression lines (solid lines), the regression slopes (s) and correlation coefficients (r). Grey dashed lines indicate the 1:1 ratio.

Fig 3 shows the scatter plots of simulated vs. observed hourly PM_2.5_ in each of the 26 YRD cities. The results show that our model moderately captured the spatial and temporal variations of PM_2.5_ in the YRD region. The correlation coefficient (r) was calculated as 0.49 (*p*-value less than 0.001) for the whole YRD region, ranging from 0.36 to 0.57 in different cities (*p*-value less than 0.001). Fig 3 also shows the regression slopes, which ranged from 0.90 to 1.20 for most cities and again verified the capability of the model in predicting the magnitude of PM_2.5_.

### Simulated PM_2.5_ in the YRD region

**Fig 4:**
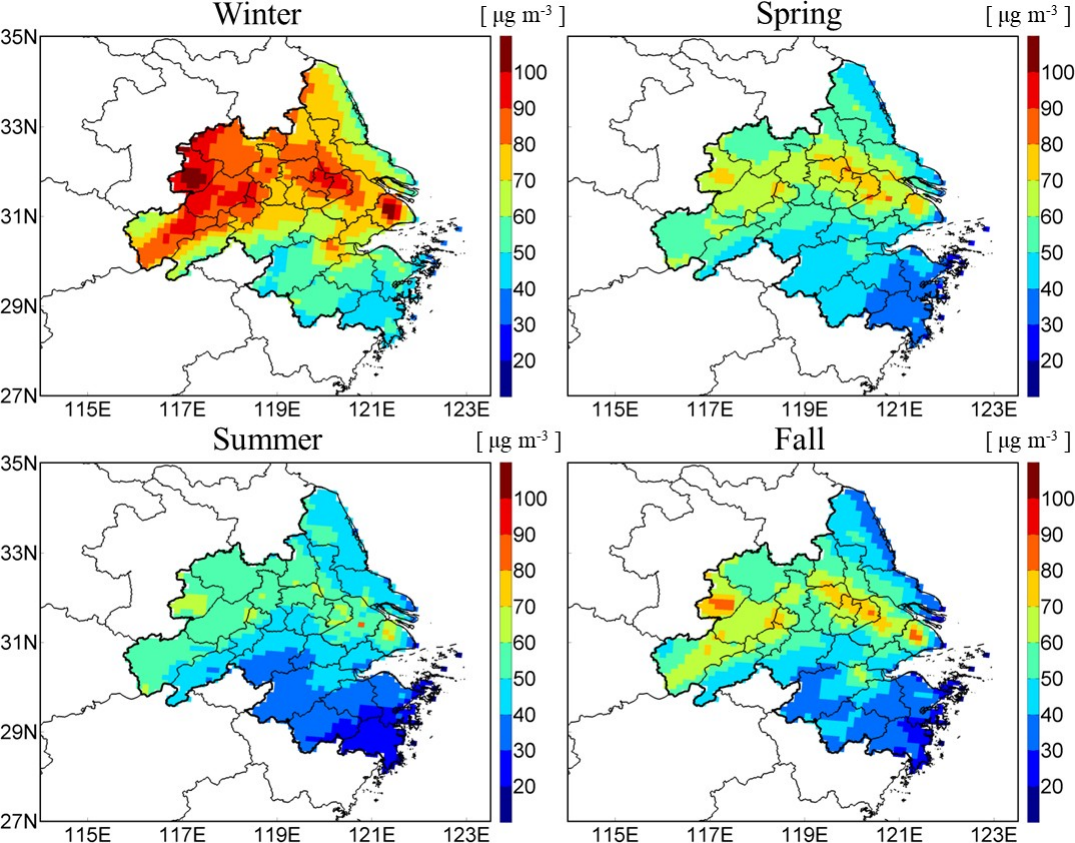
Seasonal variations of the simulated PM_2.5_ concentrations in the YRD region.

Fig 4 shows the spatial distributions of the simulated seasonal mean PM_2.5_ over the YRD region. PM_2.5_ level in winter was the highest in the year, with the simulated PM_2.5_ reaching 80–100 μg m^−3^ in Nanjing, Zhenjiang, Changzhou, Wuxi, Suzhou and all cities in Anhui. The concentrations even exceeded 100 μg m^−3^ in downtowns of Shanghai and Hefei. The phenomenon can be mainly explained by low boundary layer and abundant transported PM_2.5_ from North China by monsoon in winter. Summer was the least polluted season in the year, mainly because of high mixing height and rich precipitation. PM_2.5_ concentrations in most of the Zhejiang province were less than 40 μg m^−3^, and the areas with the concentrations of 50–70 μg m^−3^ were limited in Shanghai, Anhui and Southern Jiangsu. The spatial patterns of PM_2.5_ in spring and fall were similar. Higher PM_2.5_ concentration of 70–80 μg m^−3^ occurred in Hefei, Zhenjiang, Changzhou, Wuxi, Suzhou and Shanghai which were related to strong local emissions (see S1 Fig) [21].

**Fig 5:**
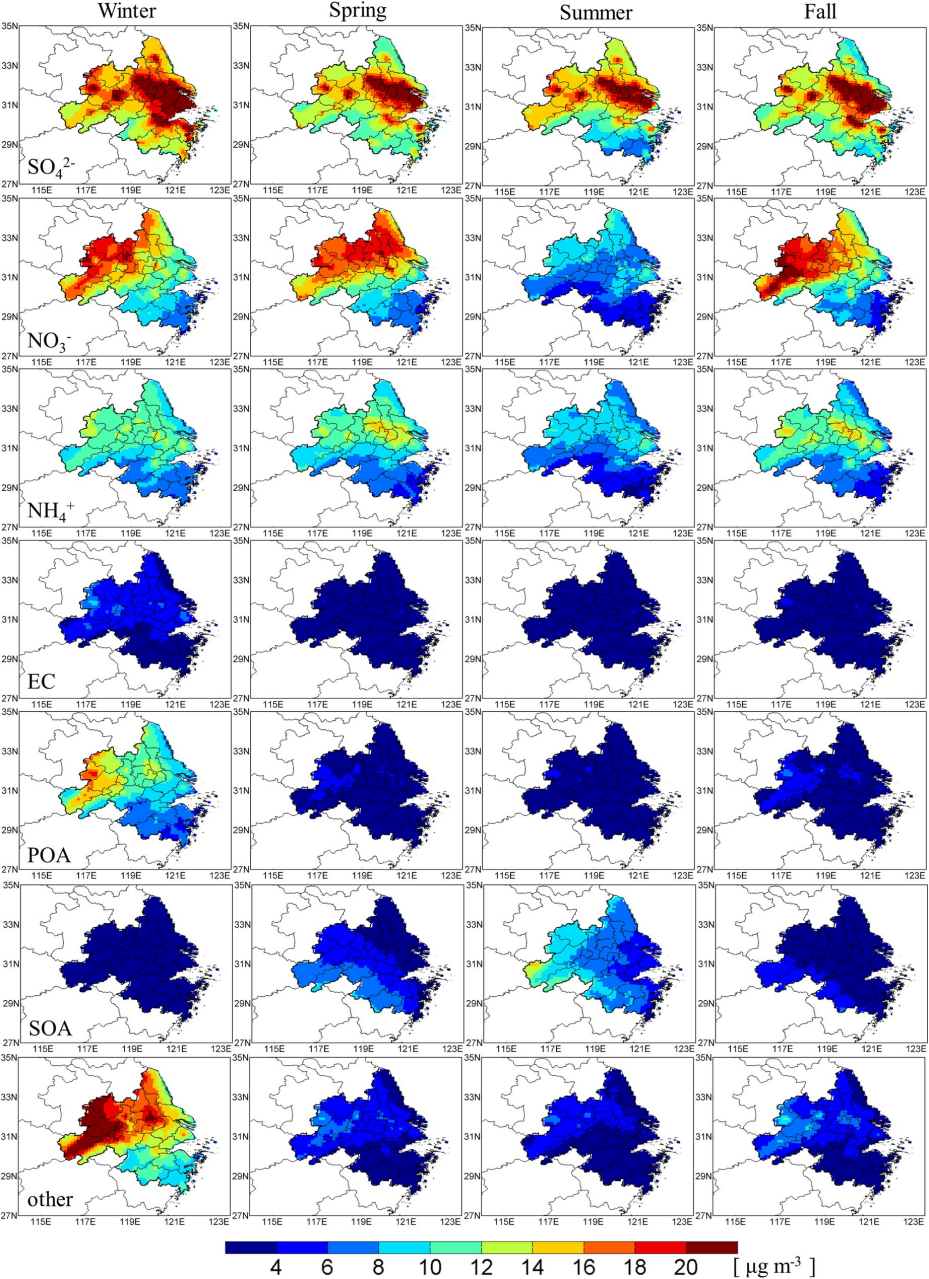
Seasonal variations of the simulated PM_2.5_ components in the YRD region.

We also analyzed the spatial distributions of seasonal mean PM_2.5_ components, including sulfate, nitrate, ammonium, element carbon (EC), primary organic aerosol (POA), secondary organic aerosol (SOA) and other components (i.e. primary PM_2.5_ mostly from dust) (see Fig 5). Secondary inorganic components (sulfate, nitrate and ammonium) and primary anthropogenic components (EC and POA) had similar seasonal variation to the total PM_2.5_, which was the highest in winter and the lowest in summer. As mentioned above, higher concentrations of secondary inorganic components and primary anthropogenic components in winter were mainly due to the lower mixing height and transport effects from North China. In contrast, SOA showed a different pattern, and the highest SOA occurred in summer. As major precursors of SOA, biogenic VOC emissions abundantly enhanced during summertime, causing the SOA concentration in summer 80% higher than the annual mean value. Nevertheless, we should note that uncertainties exist in sulfate and SOA simulations. Current models frequently underestimate sulfate especially in haze events. This may in part due to the omission of heterogeneous formation of sulfate [52,53] in chemical models. Li et al. [54] evaluated this process might contribute 20% of the sulfate in non-polluted days and the contribution would greatly enhanced as ambient PM_2.5_ increasing. In addition, current chemical models also tend to underestimate SOA due to imperfect understanding of its sources and formation pathways [55].

### PM_2.5_ concentrations from different sources

We conducted intensive sensitivity simulations to quantify the contributions of individual sources to PM_2.5_ concentration in the YRD region, by turning off the emissions from different sectors (industry, power generation, residential, transportation, and open biomass burning in the YRD region), in turn and all at once. Other simulation settings were the same as the base case.

On an annual scale, industry was the largest local source, contributing 27% to the PM_2.5_ concentration averaged for the YRD region, followed by residential (19%) and transportation activities (15%). However, the PM_2.5_ source characters exhibited remarkable variations in sub-regions in different seasons. Pie charts in Fig 1 show the corresponding contributions of each source (5 sources) to seasonal mean (4 seasons) PM_2.5_ concentrations in Shanghai, Nanjing, Hangzhou and Hefei (the capital cities of each province). Industry was the most important local source to the PM_2.5_ in all these four cities in summer (26–46%), fall (33–41%) and spring (24–30%), which was mainly due to the relative large emissions from coal-based industries in the region. In winter, the residential activities turned to be the dominant local contributor (28–45%), mostly caused by fossil-fuel and biofuel usage for domestic heating in the cold season. Transportation was also a considerable source of PM_2.5_ in the YRD region, which was the second largest source in summer (17–26%), fall (13–24%) and spring (14–18%), and even contributed 4–14% in winter. Other sources, power generation and open biomass burning, jointly contributed 3% year-round. At a city-level, contributions from industry and transportation were more obvious in Hangzhou (35% and 20%, respectively), compared with those in the other three cities (27–29% and 13–18%, respectively). Residential source played a more important role in Hefei, which was ~2 times as high as that in other cities.

In addition to the local sources, transport from outside of the YRD region (turned off the emissions from all sectors in the YRD region) also considerably contributed to the PM_2.5_ concentrations in spring (40–46%) and winter (32–44%), mainly because of the spring dust storm and winter monsoon transport, respectively. On an annual mean basis, outside sources contributed 36% to the PM_2.5_ concentration averaged for the YRD region, nearly half of the local sources’ contribution. It indicated the importance of both local emissions and external transport to the PM_2.5_ pollution in the YRD region and emphasized the necessity of regional cooperative pollution control.

### Impacts of different emission controls on PM_2.5_ reduction

We further conducted 5 scenario simulations to explore the potential impacts of emission controls on PM_2.5_ reduction. Based on the standpoint of regional cooperative control, we assumed all anthropogenic emissions in the YRD region as well as its circumjacent regions were proportionately reduced by 10%, 20%, 30%, 40% and 50%, respectively. Other simulation settings were the same as the base case.

S2 Fig compares spatial distributions of the simulated annual mean PM_2.5_ concentrations in the YRD region under different emission control scenarios, as well as the corresponding concentration changes. The PM_2.5_ concentration would effectively be reduced by mitigating anthropogenic emissions, and larger reductions tend to occur at the place of higher ambient concentrations (Shanghai, Anhui and southern Jiangsu). Table 3 shows the variations of mitigation efficiency of different schemes averaged for all YRD cities, and demonstrates an approximate linear correlation between anthropogenic emission reduction and the PM_2.5_ concentration decrease. To be specific, a 10% reduction of anthropogenic emission caused a 10% reduction of PM_2.5_ concentration. If the initial emission was reduced by 20%, 30%, 40% or 50%, the corresponding PM_2.5_ concentration would decreased by 19%, 28%, 37% or 46%, respectively. The calculated ME (as defined in Method and Data) was in the range of 0.92 to 1.00, slightly decreased as the emission reduction enhanced. The results suggested that the anthropogenic emission controls at varying degrees between 10–50% could all effectively reduce the PM_2.5_ concentration. The ME would slow down along with the declining initial anthropogenic emission, mainly because of the background PM_2.5_ concentrations and the contributions from non-anthropogenic (biogenic and open biomass burning) sources.

**Table 3:** The simulated PM_2.5_ in the YRD region under different emission control scenarios.

	Base	Scenarios				
		1	2	3	4	5
Emission reduction ratio	-	10%	20%	30%	40%	50%
PM_2.5_ concentration (μg m^-3^) ^a^	61.8	55.5	50.0	44.4	38.8	33.4
PM_2.5_ concentration reduction ratio	-	10%	19%	28%	37%	46%
Mitigation efficiency ^b^	-	1.00	0.96	0.94	0.93	0.92

^a^ The simulated annual mean PM_2.5_ concentration averaged for the 26 cities in the YRD region.

^b^ The Mitigation efficiency is defined as the PM_2.5_ reduction ratio divided by the emission reduction ratio.

## Discussion

The YRD region has been arousing more and more attention in recent year, and the heave air pollution issue manifested along with the fast economic development. We summarized the main findings of this study and proposed corresponding mitigation suggestions as follows. 1) We quantified the contribution to PM_2.5_ pollution in the YRD region was 64% from the local sources, and the rest 36% was from circumjacent regions, which emphasized the importance of both local emissions and external transport. The result highlights the necessity of regional cooperative control in environmental policy making. 2) Turning our attention to the YRD pollution, we found the dominant contributor was different from other high-polluted regions in China. In the Beijing-Tianjin-Hebei region, residential sources dominated [21], while in the YRD region we indicated industry contributed about half of the local PM_2.5_ concentration. Thus, to pointedly reduce air pollution in the YRD region, the effective method might be controlling pollutant emission from industry activities in priority order, potentially via industrial structure innovation, emission control techniques development and fossil fuels replacement [56]. Compared with residential source, industrial pollutant control is relatively more implementable, since the former includes massive emissions from necessity for human life and sometimes detrimentally from fugitive sources. 3) Our scenario simulations give us the confidence that the PM_2.5_ pollution could be effectively reduced as long as the mitigation actions bring into operation. We will always receive positive feedback from different degrees of emission controls, which is quite different with the O_3_ governance. However, this study presented a big rough picture about the pollutant gross-control in the YRD region, and the emission control scenarios were supposed to proportionately reduce the pollutant emissions from all sectors. Based on the effects of gross-control as the first step, further studies are still needed to formulate and optimize implementable mitigation actions down to sector-specific level for individual pollutants.

## Conclusion

In this study, the WRF-Chem model well captured the magnitudes and variations of observed PM_2.5_, with the normal mean biases less than ±20% for 19 out of the 26 cities from 134 ground stations. Based on the confidence built by the model evaluation, a series of sensitivity simulations were conducted to quantify the contributions from individual source sectors and from different regions to PM_2.5_ in the YRD region. The calculated results show that local sources and surrounding transport contributed 64% and 36% to the annual mean PM_2.5_ concentration, respectively, which indicated the importance of both local emissions and external transport to the PM_2.5_ pollution. Among the local sources, industry activity was the most significant sector in spring (25%), summer (36%) and fall (33%), while residential source was more important in winter (38%). We further conducted 5 scenario simulations to explore the potential impacts of varying degrees of emission controls on PM_2.5_ reduction, in which all anthropogenic emissions in the YRD region as well as its circumjacent regions were proportionately reduced by 10%, 20%, 30%, 40% and 50% respectively. The result demonstrated that regional cooperative control could effectively reduce the PM_2.5_ level. A preliminary emission control of 10% could reduce the PM_2.5_ concentration by 10%. Further emission controls would continue to reduce the concentration approximate linearly, and for places with higher ambient concentrations, the mitigation efficiency was more significant.

## Supporting information

S1 Fig Annual mean emissions of SO_2_, NO_x_, VOCs and PM_2.5_ in the YRD region for the year of 2015 from MEIC emission inventory. (TIF)

S2 Fig The simulated PM_2.5_ in the YRD region under different emission control scenarios (left panel), and the concentration changes of PM_2.5_ compared with the results of the BASE simulation (right panel).
(TIF)
