# Supplementary figures and images for: WRF-Chem modeling of particulate matter in the Yangtze River Delta region: Source apportionment and its sensitivity to emission changes
Source: PLoS One. 2018 Dec 7;13(12):e0208944. doi: 10.1371/journal.pone.0208944 (PMC6286173; doi:10.1371/journal.pone.0208944)

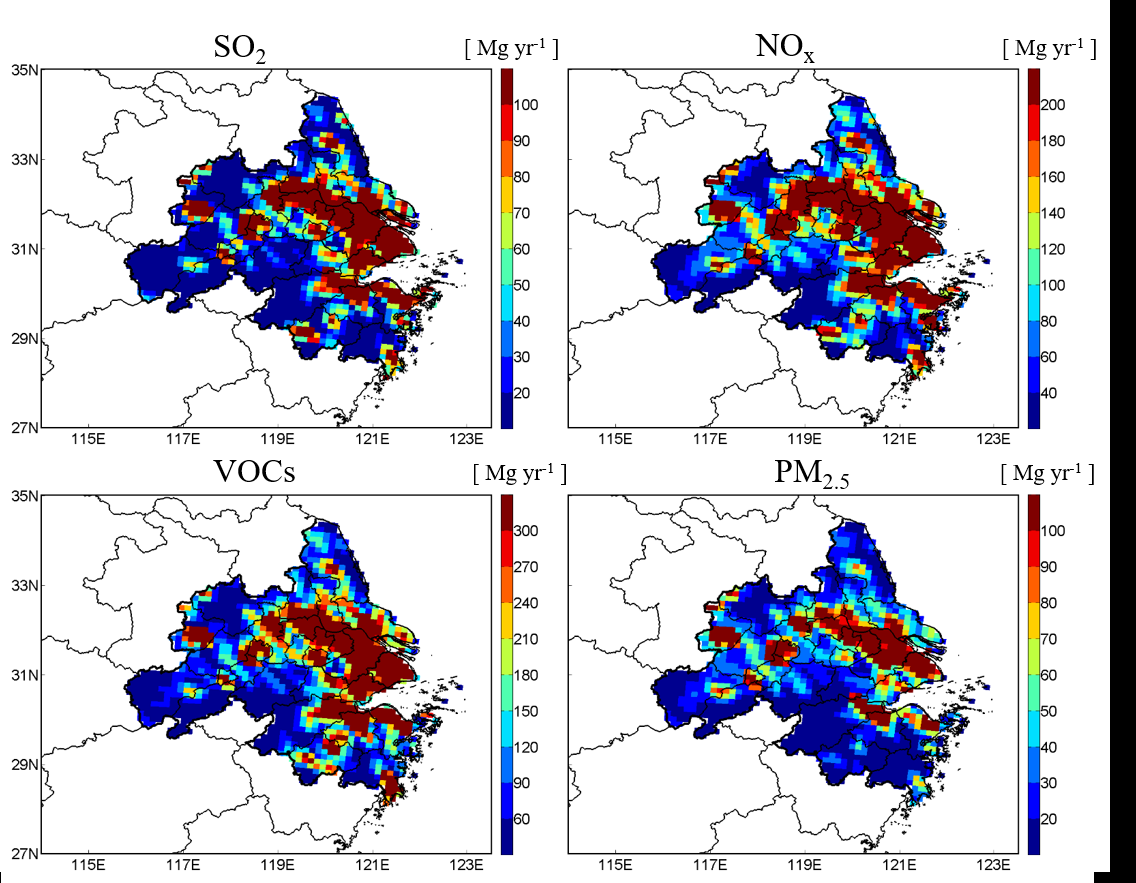

Supplement: S1 Fig — (TIF) [file pone.0208944.s001.tif]

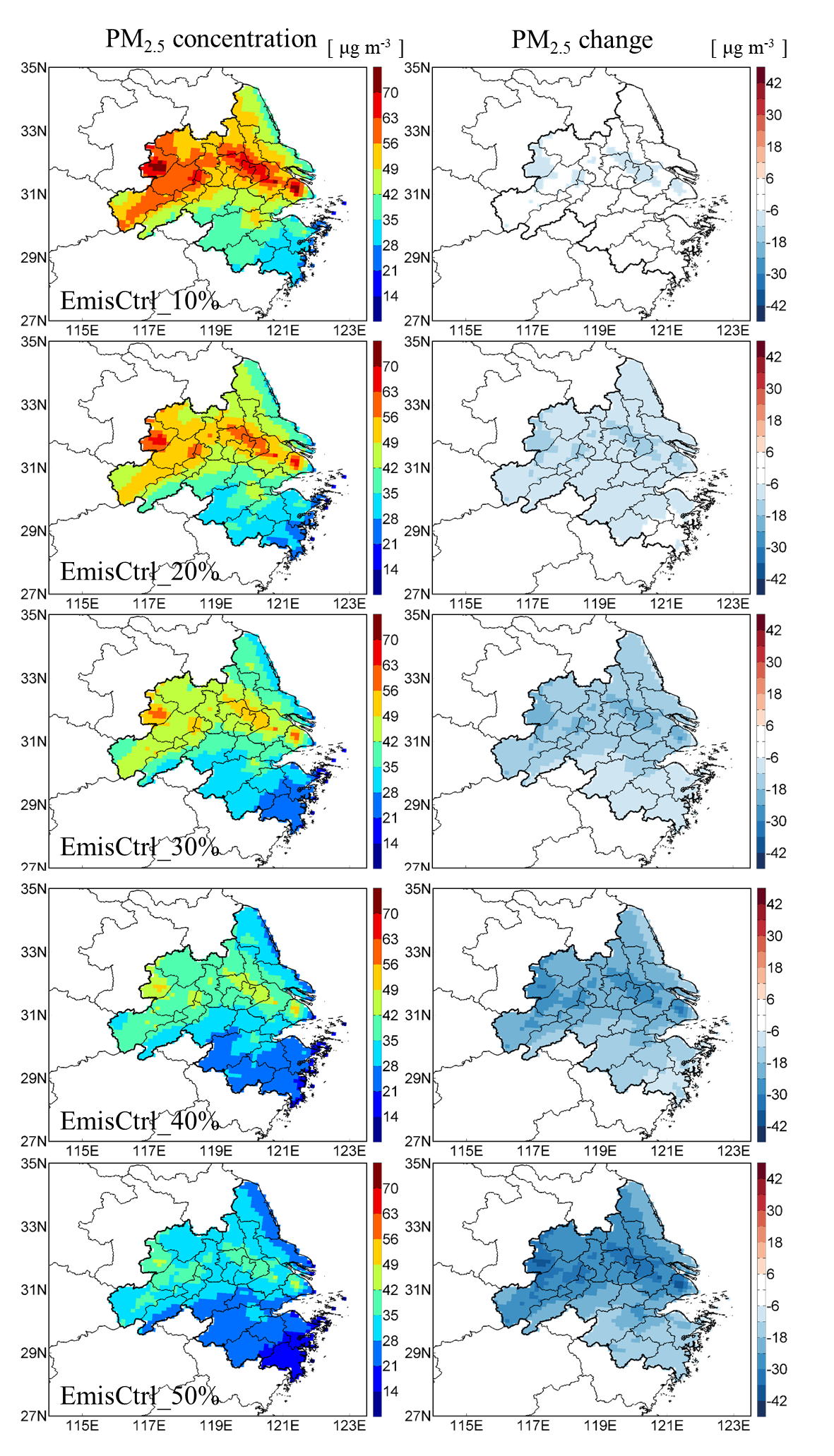

Supplement: S2 Fig — The simulated PM2.5 in the YRD region under different emission control scenarios (left panel), and the concentration changes of PM2.5 compared with the results of the BASE simulation (right panel). (TIF) [file pone.0208944.s002.tif]
